# Supplementary material for: Risk of Secondary Infection Waves of COVID-19 in an Insular Region: The Case of the Balearic Islands, Spain
Source: Front Med (Lausanne). 2020 Dec 15;7:563455. doi: 10.3389/fmed.2020.563455 (PMC7793821; doi:10.3389/fmed.2020.563455)
Supplement: Supplementary file 1 [file Data_Sheet_1.pdf]

Supplementary Material

Risk of Secondary Infection Waves of COVID-19 in an Insular Region: The Case of the Balearic Islands, Spain

Víctor M. Eguíluz et al, Front. Med. 7:563455 (2020)
doi: 10.3389/fmed.2020.563455

1 Supplementary Figures and Tables

1.1 Supplementary Figures

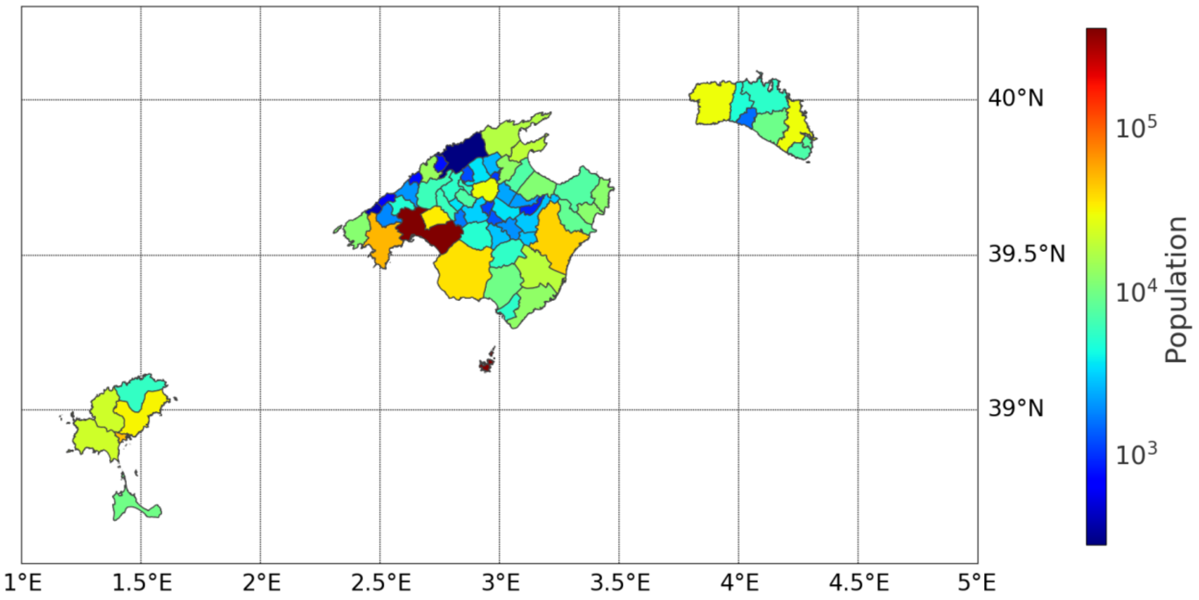

Supplementary Figure S1. Population in the 67 municipalities of Balearic Islands, according to the 2011 census. The official data does not include the small commuting flows in small municipalities, which in this case are Banyalbufar, Deià, Escorca, and Estellencs, all with less than 800 inhabitants. In this case, we have included 10 commuters from these municipalities to the neighboring municipalities and another 10 commuters to Palma, the capital and largest city of the Balearic Islands.

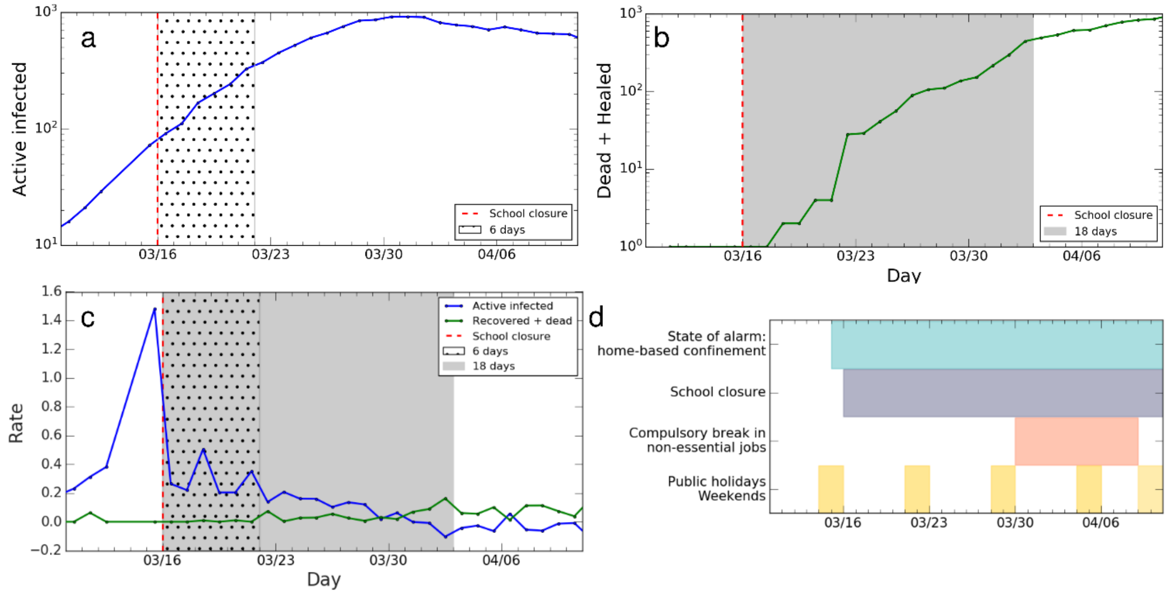

Supplementary Figure S2. Extracting the incubation and recovery times. (A,C) Daily number of confirmed cases and daily rate of change. The red dashed line indicates March 16th as the first day without schools, and the grey area covers from March 16th to March 22nd where the incidence rate starts to decay. (B,C) Daily number of confirmed healed and fatalities, and rate of change. The rate of the cases changes on April 3nd, 18 days after the school closing. (D) Main events affecting mobility and the date when they applied. The relevant periods are one day higher than the delay periods, as these delays represent the time lags without observing changes. Hence, $T_{lat}+T_{\mathrm{inf}}$=6 days, T_{dis}=12 days.

1.2 Supplementary Tables

T_{lat}	T_{inf}	T_{dis}	χ^2	α_{active}	α_{acc}	χ^2_{acc}	β_1	β_2	β_3	β_4	β_5
1	5	12	1.67	0.079	0.080	0.052	0.19	0.09	0.03	0	0
2	4	12	1.30	0.075	0.077	0.11	0.24	0.12	0.016	0.036	0
3	3	12	1.39	0.084	0.087	0.17	0.31	0.16	0.0057	0.050	0
4	2	12	1.34	0.091	0.091	0.10	0.44	0.16	0.017	0.053	0
5	1	12	1.46	0.11	0.11	0.22	0.54	0.28	0	0.52	0

T_{lat}: latent period; T_{inf}: presymptomatic infectious period; T_{dis}: disease period.

Supplementary Table S1. Accuracy of the fittings as a function of incubation, latency, and disease periods. For each set of parameters, we report the χ^2 of the model values of the number of active infected cases with respect to the official values, the correction fraction α_{active} (so that the active infected cases from the model times this factor matches the confirmed active infected cases), and the χ^2_{acc} of the model values of the number of accumulated infected individuals with respect to the official values and the correction fraction α_{acc} (so that the accumulated infected from the model times this factor matches the observed accumulated infected). For each set of parameters, the best fit is considered as the one leading to the minimum χ^2. Once the fitting values are determined, we calculate χ^2_{acc} and α_{acc}.

Date of first infection	T_{lat}	T_{inf}	T_{dis}	Prevalence (95% Confidence Interval)
Feb 7	1	5	12	4.2% (3.9 to 6.5)
Feb 7	2	4	12	3.2% (2.9 to 3.9)
Feb 7	3	3	12	2.0% (1.9 to 2.3)
Feb 7	4	2	12	3.7% (3.5 to 4.1)
Feb 7	5	1	12	2.8% (2.6 to 3.4)
Jan 28	1	5	12	25% (23 to 33)
Jan 28	2	4	12	23% (21 to 26)
Jan 28	3	3	12	16% (14 to 18)
Jan 28	4	2	12	27% (25 to 29)
Jan 28	5	1	12	22% (21 to 23)

T_{lat}: latent period; T_{inf}: presymptomatic infectious period; T_{dis}: disease period.

Supplementary Table S2. Accumulated number of infected individuals for the best fits in each iteration. Average number of total cases as the percentage of the population obtained averaging over 100 realizations, for the different sets of incubation, latency and disease periods, and date of the first infection.

2 Supplementary Text

Relation between confirmed cases and estimation cases from models

For the sake of clarity, let us assume that the number of confirmed cases grows exponentially as

$$I_c = e^{\gamma t}, \qquad \text{(S1)}$$

and that it is proportional to the real number of cases I. Thus, the time evolution of the real cases is

$$I = I_0 e^{\gamma(t+T_0)}, \qquad \text{(S2)}$$

where T_0 is the date of the first infection and I_0 is the number of initial imported cases. The imported cases are likely distributed during the time the system is open, but to illustrate our argument we will consider that all initial cases arrived the same day T_0. Thus, we can rewrite Eq. (S2) as

$$I = I_o e^{\gamma T_0} e^{\gamma t} = \alpha I_c. \qquad \text{(S3)}$$

Thus the scaling factor

$$\alpha = I_o e^{\gamma T_0}. \qquad \text{(S4)}$$

This means that the effect of the number of imported cases is additive, while the effect of the date of the first infection is multiplicative.

To check our hypothesis, we have obtained α from the growth in the number of confirmed cases, and have considered I_0 and T_0 from the data in Figure 3B. For each data point, we calculate the scaling factor as $\alpha = I_o e^{\gamma T_0}$, and plot the prevalence as a function of this theoretical scaling factor. The collapse of all the values in Fig. 3B in a single curve reflects our argument.

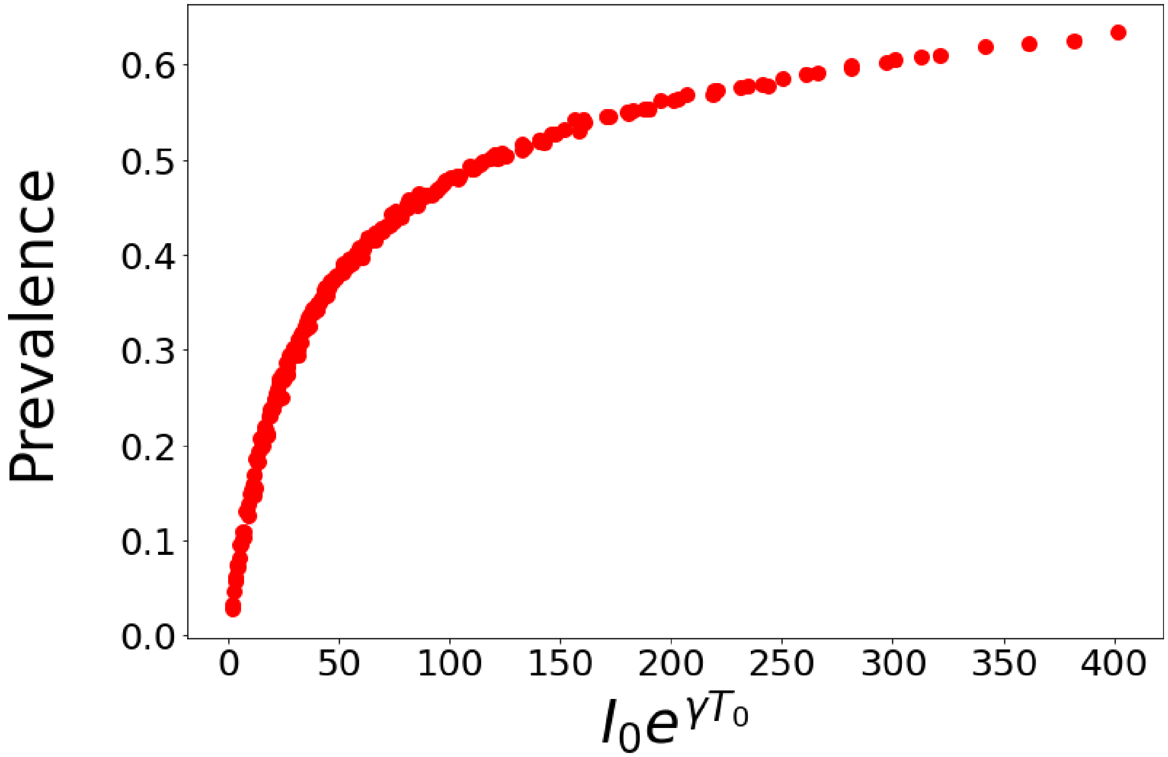

Supplementary Figure S3. Data collapse with rescaling variables. The proportion of infected cases obtained with the model follows the same curve when plotting as a function of the rescaling given by Eq. (S4) of the time of the first infection to first confirmed case T_0 and the initial number of infected individuals I_0. The data used in this figure corresponds to the data shown in Fig. 3B.
